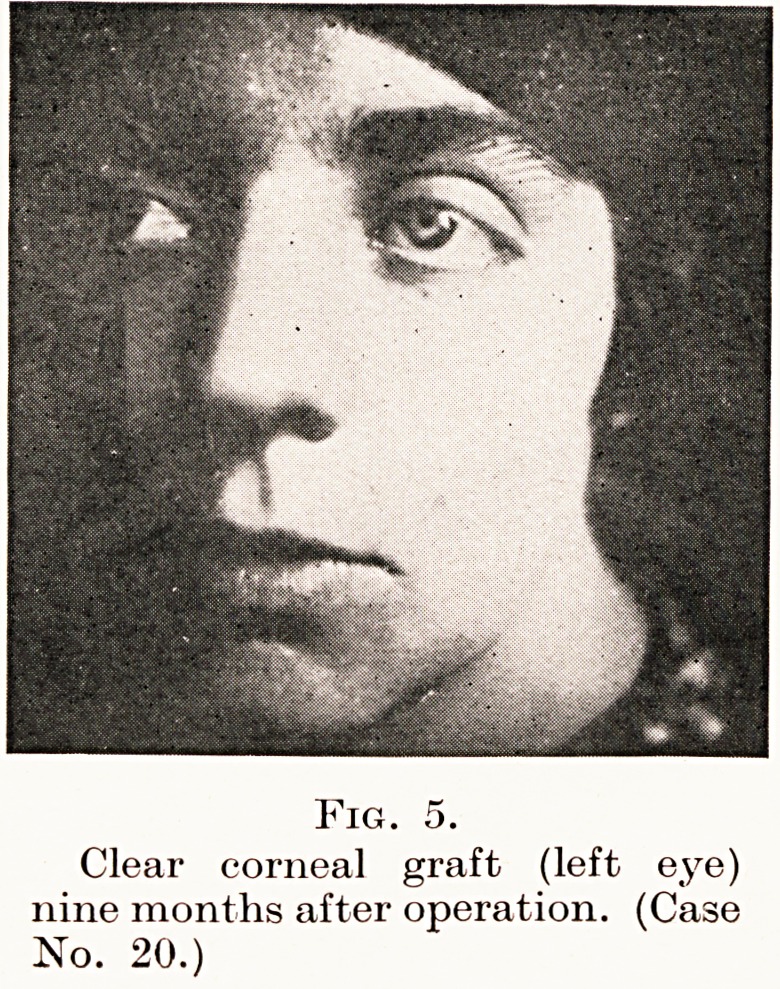# Corneal Transplantation: Its History, Development and Practice
*A Paper read before the Bristol Medico-Chirurgical Society on Wednesday, 12th February, 1936.


**Published:** 1936

**Authors:** J. W. Tudor Thomas

**Affiliations:** Honorary Ophthalmic Surgeon, Cardiff Royal Infirmary; Associate Surgeon in charge of Corneoplastic Department, Central London Ophthalmic Hospital


### The Bristol Medico-Chirurgical Journal

"*Scire est nescire, nisi id me
Scire alius sciret.*"

SUMMER, 1936.

### CORNEAL TRANSPLANTATION: ITS HISTORY, DEVELOPMENT AND PRACTICE.*

BY

J. W. Tudor Thomas, D.Sc., M.D., M.S., F.R.C.S.

*Honorary Ophthalmic Surgeon, Cardiff Royal Infirmary; Associate Surgeon in charge of Corneoplastic Department, Central London Ophthalmic Hospital.*

---

Corneal transplantation seems to have been first suggested in 1813 by Himley, and first attempted in 1818 (one hundred and eighteen years ago) by Reisinger in a rabbit. Other attempts followed, but in 1834 Dieffenbach summarized the position by stating that grafts would either fail to unite or become opaque.

In 1886 Von Hippel published the results of twelve years' work, coming to the conclusion that if the whole thickness of the cornea was removed so that

* A Paper read before the Bristol Medico-Chirurgical Society on Wednesday, 12th February, 1936.

the interior of the eye was opened the graft would become opaque owing to aqueous entering its layers. He therefore favoured an operation by which the deepest layers of the cornea were left intact and only the superficial ones removed, and claimed some success in a problem that had occupied, for sixty years, the most eminent surgeons.

In 1893 and 1894 Frohlich and Cole reported failures by Von Hippel's operation, and concluded it was not practicable to remove corneal tissue down to but not through its posterior elastic membrane—Descemet's membrane. In 1912 Magitot said that there were about half a dozen successes in corneal transplantation in the history of one hundred years. In 1921 Ebeling and Carrell reported one clear graft in a cat, and Fuchs in 1923 reported two clear grafts in twenty-eight cases.

Elschnig of Prague in 1930 published his results covering a period of twenty years using Von Hippel's trephine and a whole thickness graft. His results were a great advance on those of other workers, and he obtained 34 clear grafts out of 176 cases, but in a group of 26 cases of interstitial keratitis obtained 17 clear grafts. Twenty-three grafts failed to unite out of a group of 139 cases, and he does not state how many of the grafts in the remaining group failed to unite, but describes them as not having progressed favourably.

My own experimental work on corneal transplantation commenced in May, 1922. My interest in this subject had been aroused some time before when observing a patient blind with corneal opacities being told at an eye clinic that corneal transplantation as a means of treatment was futile. Thinking over the problem it seemed that there were many possible ways of grafting, and many factors such as size and position, or with or without conjunctival flaps, which had to be considered. With certain ideas in mind (most of which turned out to be wrong) I commenced experimental work on rabbits nearly fourteen years ago at the Physiology Institute of the Welsh National School of Medicine, and I again acknowledge my indebtedness to Professor Graham Brown, who afforded all facilities for the performance of the experiments.

The opinion of this country in regard to corneal transplantation in 1924 was summarized in Sir John Parsons' text-book on *Diseases of the Eye* in the following words: "Keratoplasty, the excision of a disc of scarred cornea and its replacement by a disc of clear cornea from a rabbit's or human eye is practically never successful. The new tissue rapidly becomes opaque."

The results of my experiments published in 1930 and 1931 were divided up into six main groups. All the grafts referred to were homologous corneal grafts in rabbits.

*The first type* (seven rabbits) consisted of grafts of cornea together with a conjunctival flap. Most of these were grafts consisting of the whole of the cornea. Four united but none was clear, although a clear area was found in some the size of which determined the size of the graft in the next group.

*The second group* consisted of central or para-central grafts (seventeen rabbits) secured in place by stitches uniting the graft with the cornea. Various devices were employed to minimize trauma to the graft by inserting stitches, but the trauma could not be dispensed with. Frequently a line of tension in the graft would cause the stitches to cut out, and seven grafts failed to unite.

*The third type.*—This group consists of seven rabbits in which no stitches were used to secure the graft in place, and the margins of the graft were cut in a shelving manner in the belief that in this way union would more readily take place on account of the increased area of the cut surfaces in apposition to each other. The graft was pressed on to the bed prepared for it, and after a few minutes' interval a drop of sterile olive oil was placed on the surface. Of the seven grafts, four became united, while three failed to unite. These seven experiments showed that a graft can adhere fairly well when the edges are cut in a shelving manner, even when no stitches are used to secure it in place, and even when olive oil is applied as a lubricant to the graft soon after the operation.

The adverse effect of an adherent iris on the transparency of a graft was also noted.

At this time an experiment was carried out in which a portion of cornea was detached except for a 2 millimetre pedicle and then sutured back in place. This united readily, and demonstrated clearly what a great difference the retention of this small pedicle made, as no clear grafts had so far been obtained.

*The fourth type.*—This group consists of eighteen rabbits, the graft in each case being taken from the margin of the cornea, triangular or quadrilateral in shape, and united to the cornea by stitches; a wide iridectomy was performed with the object in view of preventing adhesion of the iris to the graft. In thirteen of the eighteen rabbits the grafts became united, and in five they did not take. Two out of the thirteen did not live long enough for the ultimate results to be observed, but of the remaining eleven two showed a clear area for about two-thirds of the graft, while the other nine were opaque. A tendency for the stitches to produce an undesirable line of tension in the graft was again noted.

*The fifth type.*—Under this heading are included sixteen experiments. In each case the graft was secured in place by stitches inserted in the substance of the adjoining cornea and passing over the graft on its anterior surface to take a similar hold in the cornea on the opposite side, the ends then being tied together. One stitch proved insufficient, so two were used at right angles to each other. In this group one graft gave encouragement, as it exhibited a central clear area. This was the only one in which the iris was free from the commencement. The grafts in these sixteen rabbits were square or rectangular in shape, and there was a tendency for a corner of the graft to protrude. On this account the grafts in the next group were outlined with a trephine and were made round.

*The sixth type.*—In this are included four groups in which the size of the graft and gap was varied and accurately outlined by trephines of known size. It was found that the best results were obtained when the graft was cut slightly smaller than the gap in the cornea and when the margins of each were cut in a shelving manner, the graft being secured by cross stitches passing over the anterior surface in the form of a Maltese cross. The best results were obtained in a group of seven grafts all of which became united. One was opaque, one showed a small central clear area and the remaining five were transparent.

The evidence of the experiments so far was overwhelmingly in favour of this operative technique by which no stitches were inserted into the graft, the graft and corneal gap prepared by the use of trephine and scissors, with shelving margins, and the graft cut slightly smaller than the hole in the cornea. The conclusion was reached that transplantation of cornea in rabbits could be carried out with a high percentage of success.

Fig. 1 is a photograph of a clear corneal graft in a rabbit eight months after operation. The graft remained clear during the life of the rabbit for six years.

A number of lantern slides was shown at this stage indicating the details of corneal grafts in rabbits and giving details of the later results. Experimental evidence was given that heterologous corneal grafts, although they may unite, did not give the same results in regard to transparency as homologous grafts. Slides were shown demonstrating the various steps in the operative technique from the first application of the smaller trephine (to outline the graft) to the completed operation where the graft is secured in place by cross stitches which are tied over its anterior surface.

*Concerning the application of the operation to man*, a few details will be given of my first five cases, and of three other cases :—

**Case No. 1.**—My first operation for corneal transplantation on man was performed at Guy's Hospital in November, 1930, by the invitation of Mr. Ormonde.

The case was a young man whose corneæ were quite opaque due to interstitial keratitis. Two lantern slides were then shown indicating the condition of the eye one year and two years after the operation. The result in this case is that he is able to count fingers at a metre and the graft, although not quite clear, is only slightly nebulous. He is able to find his way about alone in London, and has greatly appreciated the benefit received.

**Case No. 2.**—My second case was a very unfavourable one, and the operation was only performed as rather a forlorn hope. He had suffered severely from exophthalmic goitre, had developed perforating corneal ulcers, and secondary glaucoma, and was left with very prominent eyes, staphylomatous opaque corneæ, and perception of light with a very limited field. Before grafting, the lids had to be partially secured together to narrow the palpebral aperture, because they failed to cover the prominent eye. At operation the cornea was found to be nearly 3 millimetres thick and with a totally adherent iris. Ten days after operation the graft was in place and clear, but on the thirteenth day it became detached—following closely on the news which reached him of his father's death. The hole in the cornea closed up in a month, and the eye was not lost.

**Case No. 3.**—My third case (operated on at Moorfields by invitation of Miss Mann) was again an unfavourable one—a staphylomatous cornea in a baby of 2½ years, the result of perforating ulcers accompanying ophthalmia neonatorum. The lens was absent and the iris totally adherent to the cornea. The graft united well and remained practically clear for two months, when the original dry condition of the eye caused epithelial thickening which ultimately rendered the graft useless.

Although a disappointing case, it was encouraging to know that it could be performed with success on a baby—contrary to the teaching of Elschnig, who said his operation should never be attempted under fourteen years of age.

**Case No. 4.**—My fourth case (operated on at Moorfields by courtesy of Sir John Parsons) was on a young man of 28, blinded by severe sulphuric acid burns. This eye exhibited loose conjunctival tissue on the cornea, and the eye was not really a favourable one. The graft united but became opaque. The procedure was repeated with the same result two years later, and I performed a third grafting operation on him at the Royal Westminster Ophthalmic Hospital, by kind invitation of the Honorary Staff, five weeks ago. It is possible that the last operation may give him a little benefit, but the unfavourable condition of his eye is again asserting itself, and I am very doubtful about it.

**Case No. 5.**—My fifth case (operated on at Moorfields by courtesy of Mr. Whiting) was quite a suitable case, and the graft united perfectly and was quite transparent, but nevertheless it is a sad story. During the third week after operation he developed signs of acute abdominal trouble, with vomiting and backache, and began to vomit blood. He was transferred to Middlesex Hospital with a diagnosis of gastric ulcer. Mr. Gordon Taylor performed a laparotomy and found an unsuspected but quite inoperable carcinoma of the stomach. He died exactly three weeks after the corneal operation with a perfectly successful and transparent corneal graft. The photograph (Fig. 2) shows the condition of the eye post-mortem.

I will mention very briefly three more cases :—

**Case No. 11.**—Miss K., a young Canadian lady of 34, blind with opaque corneæ from silver nitrate applications as a baby of two weeks' old. Before operation her vision was hand movements only. Four and a half months after operation she became able to count fingers at 18 in. She had never known what it was to see things in the ordinary way, and it would take much more time than is now available to give the extremely interesting details of this case. She became able to read writing on the blackboard in 2 in. letters, at the rate of about one letter a second—at a distance of 6 in. to 8 in. from the board. This case was demonstrated by her own ophthalmic surgeon, Dr. Conrad Berens of New York, at the New York Ophthalmological Society in November, 1934.

**Case No. 12.**—Mr. J. B., a New Zealander, age 59, with extensive corneal opacities from ulceration twenty and forty years previously. Four months after operation the graft was clear with a small opacity behind it near the lower part of the graft. His vision improved to 6/36, and he enjoyed seeing London and was able to go about alone. On the eve of his departure home to New Zealand six months after operation he developed an acute illness from which he died.

**Case No. 20.**—V.T., a young lady of 22, is one of my cases that I have never demonstrated before, and I am particularly glad that she was able to come here to be demonstrated this evening because she is a girl from this locality. I first saw her in May, 1934, and Dr. Cyril Walker very kindly provided me with useful information as to her past history. She developed severe interstitial keratitis at the age of ten, and was treated at the Bristol Eye Hospital for two years. She

PLATE VII

Fig. 1.
Clear corneal graft in rabbit eight months after operation.

Fig. 2.
Transparent corneal graft three weeks after operation. (Case No. 5.)

Fig. 3.
Left eye opaque cornea before operation. (Case No. 20.)

Fig. 4.
Clear corneal graft nineteen days after operation. (Case No. 20.)

Fig. 5.
Clear corneal graft (left eye) nine months after operation. (Case No. 20.)

was left with extensive and dense corneal opacities, as may be seen in the photograph (Fig. 3) of the left eye taken on 14th December, 1934. The right eye was able to count fingers no more than 4 in. away, and the left eye could only see hand movements. Corneal transplantation was performed on the left eye on 15th December, 1934, fourteen months ago. She was an ideal patient, calm, thoughtful and intelligent, and she did very well. Exactly nineteen days after the operation another photograph was taken, which is reproduced herewith. (Fig. 4.) Nine months after operation (Fig. 5) the graft was still clear, and her vision was 6/36 partly. The reason why her visual acuity is not much better is that she has traces of cataract following the original severe inflammatory disease which caused her blindness.

She has written me many letters to keep me informed of her progress. Three months after her operation I was pleased to receive a letter from her beginning: "I am taking the pleasure of writing you this short letter all by myself," and ending with expressions of gratitude.

She has in the last few months been able to read many books, and tells me she read a book with good print of 237 pages in four nights. She can read numbers in a telephone directory.

It is now fourteen months since her operation, and you have had an opportunity of seeing the condition of the eye yourselves.

The results of a series of corneal grafts were then given, and compared with those of other workers on this subject.

At the time of giving this address I have performed operations of corneal transplantation on the human subject thirty-four times, two of them as recently as four days ago. As far as I can judge, excluding the two recent cases, the percentage of successful results giving a definite improvement in vision with a graft which retains much of its transparency is over 70 per cent. Minor modifications of technique are being put into practice according to the deductions from clinical and experimental work, so that as far as possible a rational line of thought is maintained, and patients are not subjected to an unjustifiable departure from the established technique. The effect of these small alterations in technique is carefully observed, and in course of time the modification is accepted or rejected. At the present time it has been established that suitable cases can be treated with good prospect of benefit by corneal transplantation.

The most suitable type of case is one where the eye is practically normal with the exception of the presence of an opaque cornea. Such a case may occur as a result of inherited syphilis. It is possible to obtain accurate knowledge of the presence and depth of the anterior chamber, the position, size and reaction of the pupil, in a case of opaque corneæ by double transillumination. The function of the retina is estimated by testing the patient's projection of light. It is palpably useless to operate on an opaque cornea if there is optic atrophy and the eye cannot perceive light. Careful subjective testing can determine in most cases to what extent the retinal function is diminished or whether it is about normal. It is not infrequently found after perforating ulcers leaving a dense corneal scar that a condition of chronic secondary glaucoma with increased tension of the eye has gradually limited the field of vision to a small central area, or has caused total blindness, in which case an operation is not indicated.

In considering the suitability of an eye for operation note has also to be taken of the condition of the conjunctiva, the presence of a band of loose tissue on the cornea, adhesion of the eye to the lids, and the general cleanliness of the eye. Some of these conditions can be treated by preliminary operations and medical treatment, so that an unsuitable eye may be converted into an eye reasonably suitable for grafting.

In approaching these cases, therefore, I have to ask myself not only, "Is this eye suitable for operation ?" but, "Can the eye be made reasonably suitable for operation by any possible means of preliminary treatment ?"

It sometimes happens that a hopeless-looking eye, which at a first glance one would dismiss as quite unsuitable, can be so improved as to render it suitable.

Quite recently I saw such a case, following a severe burn ; treatment of that case, at present hopelessly blind, will probably take about three years and will mean several operations, but in time I believe he will be enabled to take a chance of getting some useful vision with a reasonable prospect of success. I have seen many cases with opaque and scarred corneæ in which the eyes are otherwise diseased or damaged in such a way as to afford practically no chance of success in the present state of our knowledge. To these problems I am devoting much of my time and thought, and no doubt others are doing so too or will do so. One distinguished friend and colleague told me that the outlook of these cases would be just as bad in future as it is now, as he did not see how the present difficulties could be overcome. My own view is very much the reverse, I believe that many of the difficulties at present apparently insuperable can be overcome by patience, perseverance and constant application to the problem. In this way, imbued with a spirit of hope and optimism, progress can be made, and many of the difficulties of one generation do not exist in the experience of the next. That the next generation will have some difficulties handed on to them for solution is probable; that they will encounter fresh difficulties and still more difficult problems to be solved is almost certain; that these arrangements are a kind dispensation of Providence is not improbable, for the benefit of humanity and the training of mankind.

## Figures and Tables

**Fig. 1. f1:**
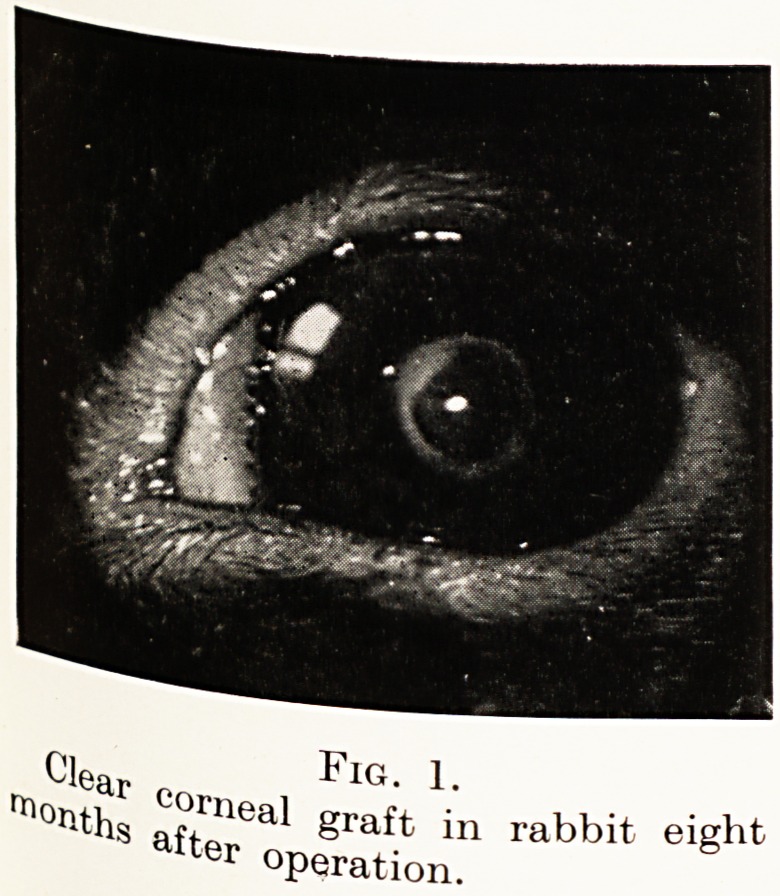


**Fig. 2. f2:**
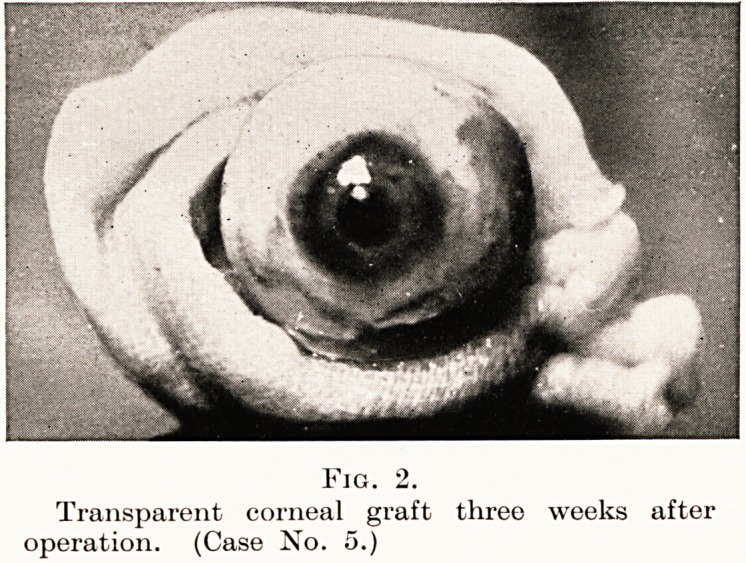


**Fig. 3. f3:**
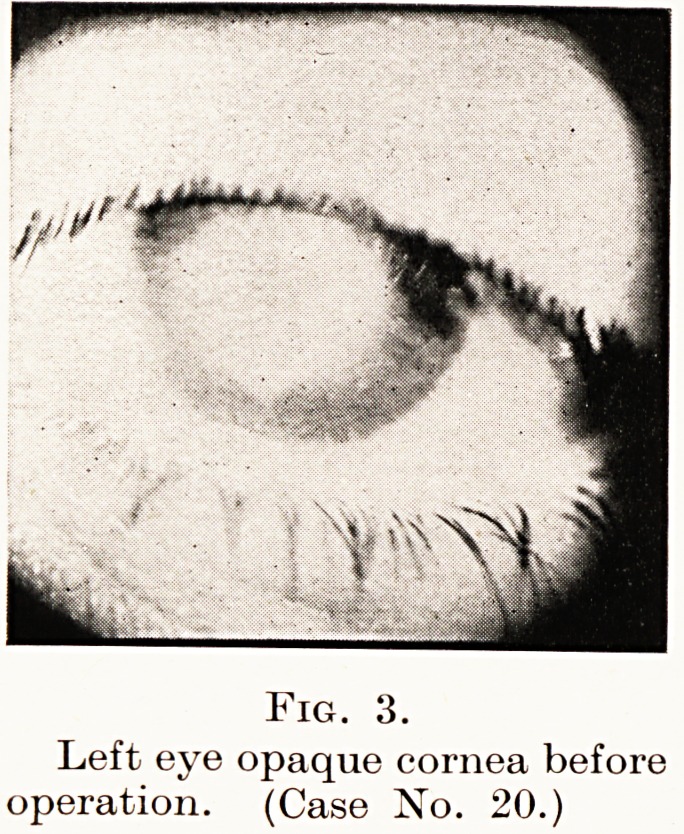


**Fig. 4. f4:**
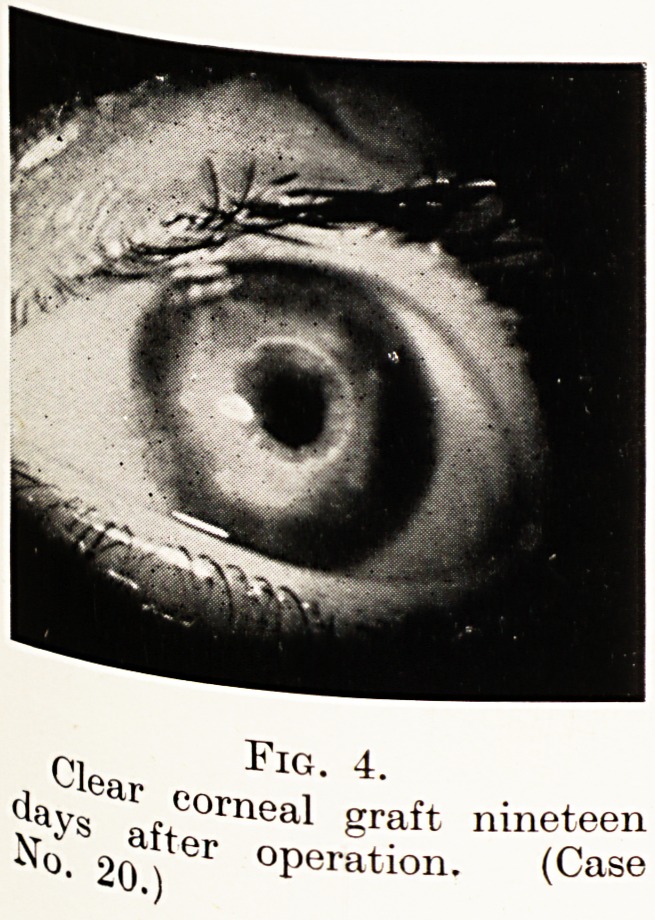


**Fig. 5. f5:**